# Molecular Dipoles as a Surface Flattening and Interface Stabilizing Agent for Lithium‐Metal Batteries

**DOI:** 10.1002/advs.202301426

**Published:** 2023-05-23

**Authors:** Seo‐Young Jun, Kihyun Shin, Jun‐Seo Lee, Suji Kim, Jinyoung Chun, Won‐Hee Ryu

**Affiliations:** ^1^ Dept. of Chemical and Biological Engineering Sookmyung Women's University 100 Cheongpa‐ro 47‐gil Yongsan‐gu Seoul 04310 Republic of Korea; ^2^ Dept. of Materials Science and Engineering Hanbat National University Daejeon 34158 Republic of Korea; ^3^ Emerging Materials R&D Division Korea Institute of Ceramic Engineering and Technology (KICET) Jinju 52851 Republic of Korea; ^4^ Institute of Advanced Materials and Systems Sookmyung Women's University 100 Cheongpa‐ro 47‐gil Yongsan‐gu Seoul 04310 Republic of Korea

**Keywords:** dendrite suppression, electrolyte additive, lithium‐metal anodes, *N*‐methyl‐2‐pyrrolidone, surface leveler

## Abstract

Reaching the border of the capable energy limit in existing battery technology has turned research attention away from the rebirth of unstable Li‐metal anode chemistry in order to achieve exceptional performance. Strict regulation of the dendritic Li surface reaction, which results in a short circuit and safety issues, should be achieved to realize Li‐metal batteries. Herein, this study reports a surface‐flattening and interface product stabilizing agent employing methyl pyrrolidone (MP) molecular dipoles in the electrolyte for cyclable Li‐metal batteries. The excellent stability of the Li‐metal electrode over 600 cycles at a high current density of 5 mA cm^−2^ has been demonstrated using an optimal concentration of the MP additive. This study has identified the flattening surface reconstruction and crystal rearrangement behavior along the stable (110) plane assisted by the MP molecular dipoles. The stabilization of the Li‐metal anodes using molecular dipole agents has helped develop next‐generation energy storage devices using Li‐metal anodes, such as Li–air, Li–S, and semi‐solid‐state batteries.

## Graphical abstract

This study reports a *N*‐methyl‐2‐pyrrolidone (MP) molecular dipoles as an anode surface flattening and interfacial stabilizing agent to achieve longer lifespan and high stability Li‐metal batteries. The simple process of dissolving the MP into the electrolyte offers advantages including a dendrite suppression, modulation of chemical components in solid electrolyte interface layer and crystal rearrangement during Li deposition, thereby enhancing cell stability.

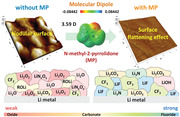

## 1 Introduction

Boosting the energy density of existing Li‐ion batteries (LIBs) is required in order to realize smaller‐sized portable electronics, long‐range electric vehicles, and large‐scale energy storage systems.^[^ ^1^ ^]^ Currently commercialized LIBs have been widely used in the battery market, yet the theoretical specific capacity of a conventional graphite anode (≈370 mAh g^−1^) cannot satisfy the growing demand for energy density.^[^ ^2^ ^]^ Li metal has received a lot of research attention as an ideal anode due to its undoubtedly attractive merits, such as exceptional theoretical capacity (≈3800 mAh g^−1^), low weight (0.53 g cm^−3^), and lowest electrochemical potential (−3.04 V vs standard hydrogen electrode).^[^ ^3^ ^]^ Nevertheless, it is still difficult to overcome the intrinsic problems of Li‐metal anodes toward the commercialization of Li‐metal batteries. Highly reactive Li metal causes inevitable side reactions in most electrolytes.^[^ ^4^ ^]^ In addition, Li dendrites are formed toward the counter cathode during repeated cycling, which causes a short circuit and safety issues. Dead Li accumulation and the formation of numerous pores in the Li metal during cycling gradually deteriorate the coulombic efficiency and volumetric energy density of Li‐metal batteries. Huge volume changes that induce mechanical stress are also present in the cell, and other unwanted reactions occur during the lithium deposition and stripping processes.^[^ ^5^ ^]^

The severe issues of Li‐metal anodes usually originate from dendrite formation caused by the non‐uniform and heterogeneous deposition of Li on the Li‐metal anode during repeated charge/discharge processes. Various strategies have been used to alleviate these issues in order to enable a uniform Li‐ion distribution, including i) modifying the electrolyte composition, ii) introducing stabilizing electrolyte additives, iii) engineering an artificial solid electrolyte interface (SEI) layer, and iv) constructing 3‐D current collector host materials for Li metal.^[^ ^6^ ^]^ Among these strategies, those that provide stabilization using electrolyte additives offer attractive advantages due to their controllable functionality, simplified process, and efficiency. Diverse electrolyte additives, such as phosphates, inorganic salts, and fluorinated molecules, have been applied to effectively control the SEI layer component or stabilize the surface by controlling the deposition behavior of Li ions.^[6a,6c,7]^ For example, fluoroethylene carbonate (FEC) is a representative electrolyte additive used for Li‐metal battery systems that can easily generate a relatively strong and stable LiF‐rich SEI layer on the Li‐metal anode.^[^ ^8^ ^]^ Although modifying the composition of the SEI layer using additives has been successful, the random growth and formation of problematic Li dendrites should be addressed. Therefore, it is necessary to develop an additive alternative that simultaneously exhibits a dendrite inhibitory effect and regulates the SEI layer components to improve the performance.

**Figure 1:**
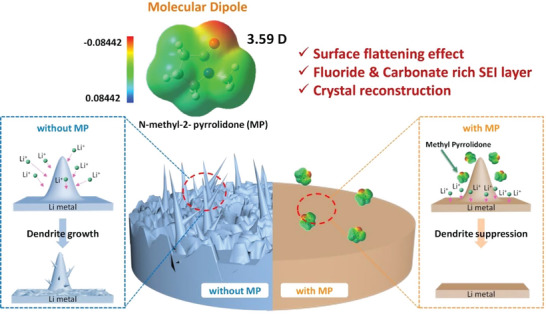
A schematic illustration of MP molecules used as a surface leveling additive in Li‐metal electrodes.

To obtain a better and more compatible solution, we have revisited pyrrolidone‐based surface‐leveling additives that have been used to flatten the electrodeposition layer in the classical electroplating technique. In this study, we introduce *N*‐methyl‐2‐pyrrolidone (MP) molecular dipoles into the electrolyte as a surface leveler and SEI layer transformer for longer cycling and highly efficient Li‐metal batteries. The MP molecular dipoles successfully regulate the Li deposition behavior and achieve a stable and flat surface morphology without dendrite formation on the Li‐metal electrode during repeated deposition and stripping processes. When an electric field is applied at the electrochemical interface, a biased electron distribution on the heterogeneous surface can be counter‐balanced by adsorbing MP molecules with a permanent dipole moment (3.59 D) and polarizability (10.6 ± 0.5 10^−24^ cm^3^), thereby enabling the uniform and flatter deposition of the Li‐metal electrode without dendrite formation (**Figure** **1**). Using a Li–Li symmetric cell test, the effect of MP on the long‐term surface stabilization of a Li‐metal electrode even at a high current density of 5 mA cm^−2^ was demonstrated in tetraethylene glycol dimethyl ether (TEGDME) electrolyte. We controlled the concentration of the MP molecular dipoles in the electrolyte and elucidated the importance of an appropriate amount of MP. Furthermore, we confirmed (i) the formation of a stable SEI layer containing durable species and (ii) the modification of the crystalline growth behavior using ex situ analysis. Density functional theory (DFT) calculations were performed in order to better understand the change in the binding energy of Li observed upon adding MP molecular dipoles and to support the dendrite suppression and surface reconstruction observed experimentally. Our work suggests a potential solution for overcoming the disadvantages of Li‐metal batteries by choosing suitable molecular dipoles that enable surface leveling and SEI stabilization for Li electroplating and stripping reactions to realize high energy Li‐metal battery systems.

## 2 Results and Discussion

### 2.1 Li–Li Symmetric Cells Employing MP Molecular Dipoles

We examined the potential of N‐methyl‐2‐pyrrolidone (MP) molecular dipoles as an electrolyte additive to achieve i) dendrite suppression, ii) stable SEI layer formation with durable components, and iii) crystalline structural rearrangement of highly stable and long‐life Li‐metal anodes. MP additives, a family of pyrrolidone‐based monomers, can stabilize the surface energy by adsorbing dipolar MP molecules on nodular and unstable reaction sites, thereby leveling the Li metal surface. Therefore, MP dipoles effectively inhibit dendrite growth on the surface during the Li deposition process (Figure 1).

**Figure 2:**
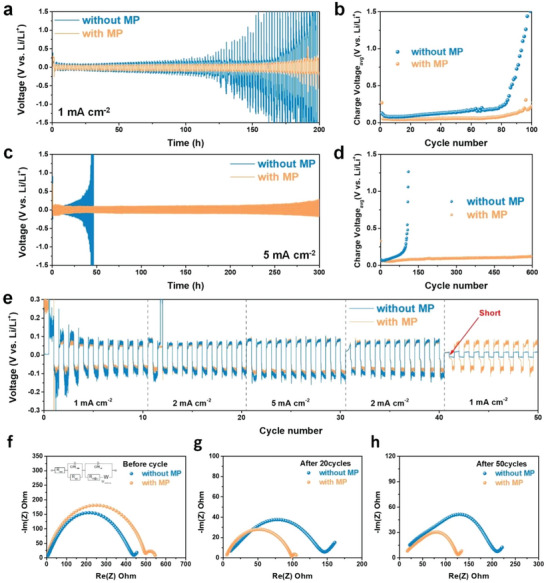
Li‐Li symmetric cell tests using an electrolyte prepared with and without MP. Cell performance tests of the lithium metal electrodes performed a) at a low current density of 1 mA cm^−2^ depicted as the charge/discharge curve and b) the average charge voltage, c) performed at a high current density of 5 mA cm^−2^ depicted as the charge/discharge curve and d) the average charge voltage. e) Rate performance test using Li–Li symmetric cells prepared with and without the MP additive. EIS spectra obtained for the Li–Li symmetric cells prepared with and without MP additive: f) Prior to cycling, g) after 20 cycles, and h) after 50 cycles.

Cycling tests using of Li–Li symmetric cells with MP molecular dipoles in the electrolyte were conducted at different current densities in order to prove their effect (**Figure** **2**). The increase observed in the overpotential during the symmetric cell test results corresponds to cell degradation due to (i) excessive electrolyte consumption, ii) the formation of an unstable and resistive SEI layer on the Li metal surface, iii) the formation of dead Li, and iv) short circuit due to Li dendrites.^[^ ^4^, ^9^ ^]^ Figure 2a presents the voltage‐time profiles obtained for the Li–Li symmetric cells constructed with and without MP molecular dipoles. Voltage fluctuations and increased overpotentials were observed for the pristine Li–Li symmetric cells after 100 h. In contrast, Li–Li symmetric cells with MP exhibit two‐fold stable cycles over 200 h without any notable increase in the overpotential at a current density of 1 mA cm^−2^. Upon comparison of the results obtained after 150 h, a large overpotential difference was observed between the cells constructed with and without MP molecular dipoles, which was associated with the degree of non‐uniform deposition and fast electrolyte consumption on the surface of the Li‐metal electrode (Figure S1a, Supporting Information).^[^ ^6^, ^9^, ^10^ ^]^ The average charge voltage of the Li–Li symmetric cells without MP, which is related to the increased overpotential, was substantially increased after 80 cycles. In contrast, Li–Li symmetric cells with MP were successfully stabilized for up to 100 cycles (Figure 2b). The effect of the MP molecular dipole additive was confirmed more clearly at a high current density of 5 mA cm^−2^. Although the cells without MP started to degrade after 46 h, the MP‐containing Li–Li symmetric cells exhibited lower voltage polarization without voltage fluctuation and exceptional cycle performance even for 300 h during repeated Li plating and stripping (Figure 2c,d). The stabilizing effect was apparently reinforced in the presence of MP dipoles, as shown upon comparing the voltage‐time profiles of the cells obtained at a higher current density of 5 mA cm^−2^ (Figure S1b, Supporting Information). In this regard, the average charge voltage values (≈0.1 V) of the Li–Li symmetric cells prepared with MP were stably maintained over 600 cycles when compared to the pristine cell (<108 cycles). The results demonstrate that the preferred nucleation and subsequent dendrite formation were effectively inhibited by the MP molecular dipoles, especially under high rates of Li plating and stripping, which correspond to fast charging and discharging. The voltage polarization curves obtained for the Li–Li symmetric cells prepared with and without MP every ten cycles at different current densities are presented in Figure 2e. Both the cells with and without the MP additive showed smooth changes in the voltage polarization for the initial cycles. The cell without MP started to exhibit voltage fluctuation peak after ten cycles at current density of 2 mA cm^−2^. In contrast, no significant voltage fluctuation was observed in the Li–Li symmetric cells prepared with MP during the entire cycle. Moreover, when the current density was changed from 2 to 1 mA cm^−2^, the voltage was not stable in the cell without the MP additive due to the short circuit. As the dendrites penetrate the separator and reach the opposite cathode, a voltage close to 0 V is associated with cell failure.^[^ ^11^ ^]^ MP molecular dipoles can successfully stabilize the Li metal surface without significant dendrite growth or short‐circuit issues in the cell.

Electrochemical impedance spectroscopy (EIS) tests using the Li–Li symmetric cells prepared with and without MP were performed before and after the 20^th^ and 50^th^ cycles in order to investigate the charge transfer kinetics and interface resistance observed between the Li‐metal anode and electrolyte, (Figures 2f–h). The equivalent circuit used in the Li–Li symmetric cell tests is shown in the inset of Figure 2f. The corresponding *R*_sol_ and *R*_ct_ values of the cells are summarized in Table S1 (Supporting Information). *R*_sol_ is associated with the ohmic resistance, which is the sum of the resistive components due to the migration of Li ions through the interface between the electrolyte, separator, and Li‐metal electrode.^[^ ^12^ ^]^ The ohmic resistance is significantly affected by the reaction at the electrolyte/electrode interface. When a battery deteriorates, a side reaction due to electrolyte decomposition occurs, resulting in a decrease in the Li ion conductivity and an increase in the ohmic resistance.^[12b]^ Both the pristine and MP‐containing cells show an increasing trend in the ohmic resistance as the cycle progresses, indicating that the movement of the Li ions was impeded during the cycling process. However, when comparing the *R*_sol_ value after the 20^th^ and 50^th^ cycles, it can be observed that a more stable interfacial resistance was maintained in the presence of the MP additive in the electrolyte. In addition, the size of the arc in relation to the charge transfer resistance (*R*_ct_) in the EIS graph is a noteworthy feature. Two semicircles formed at high frequency related to the *R*_ct_ between the electrolyte/SEI layer and SEI layer/Li‐metal electrode, which can be associated with the diffusion of Li ions.^[^ ^12^, ^13^ ^]^ Prior to cycling, the R_ct_ value (498.05 Ω) in case of the cell prepared with MP were larger than that observed for the cell without MP (438.79 Ω) because the absorption of the MP molecular dipoles at the electrochemical reaction sites may disturb the charge transfer between the electrolyte and Li‐metal electrode (Figure 2f).^[13b]^ Nevertheless, the tendency observed for *R*_ct_ between the different cells was reversed after the cycling progressed. After cycling, the *R*_ct_ observed for the MP‐containing cell exhibited a much smaller value (99.05 Ω) than that for the pristine cell without MP after the 20^th^ cycle (146.03 Ω), as shown in Figure 2g. After the 50^th^ cycle, the *R*_ct_ of the cell prepared with MP cell was successfully stabilized at 126.73 Ω when compared the reference cell (211.16 Ω), as shown in Figure 2h. The low *R*_ct_ value observed for the MP‐containing cell indicates fast electrochemical kinetics and surface stabilization originating from rapid charge transfer and controlled dendrite growth during Li stripping and plating, respectively. Figure S2a (Supporting Information) shows the total cell resistance as a function of the number of cycles. Although a slight increase in the cell resistance was observed due to the surface absorption of MP molecules prior to cycling, it can be concluded that the overall resistance of the cell was reduced upon introducing the MP molecular dipole additive into the electrolyte.

### 2.2 Concentration Effect of the MP Additive

**Figure 3:**
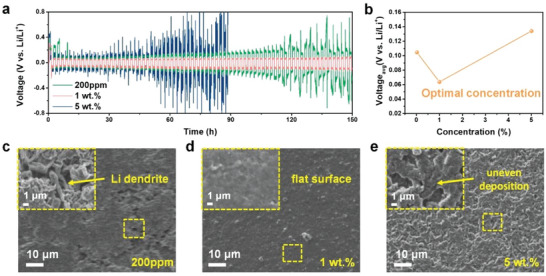
Effect of the MP additive concentration in the electrolyte. a) Voltage time profiles obtained for the Li–Li symmetric cell at a low current density of 1 mA cm^−2^ using three different concentrations of MP additive. b) Optimal concentration of the MP containing electrolyte. SEM images of Li deposited on Cu foil at the current density of 0.5 mA cm^−2^ for 2 h using c) 200 ppm, d) 1 wt.%, and e) 5 wt.% MP.

**Figure 4:**
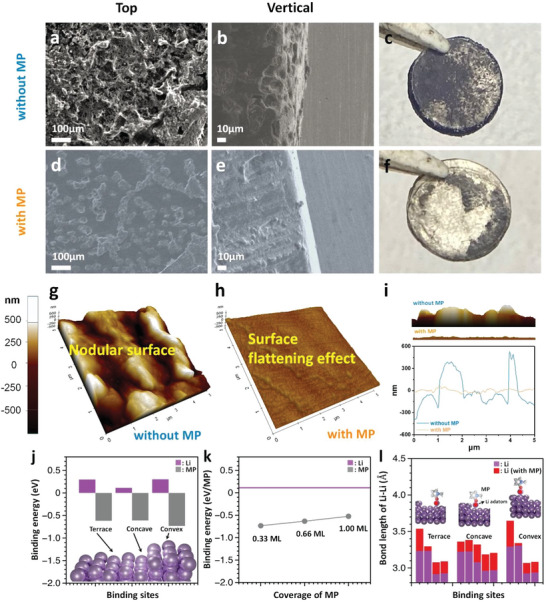
Ex situ surface morphology of the Li–Li symmetric cell observed at a current density of 5 mA cm^−2^. The SEM images of the Li‐metal morphology after 500 cycles at a current density of 5 mA cm^−2^ in the electrolyte with and without MP: a) top and b) cross‐sectional view using an electrolyte without MP, and d) top and e) cross‐sectional view in the presence of MP. Digital images of the Li‐metal electrode from the disassembled Li–Li symmetric cells prepared c) without MP and f) with MP. AFM images of the Li‐metal electrode after 500 cycles at a current density of 5 mA cm^−2^ in the electrolyte g) without MP and h) with MP, and i) cross‐sectional graph with and without MP. Binding energies of Li metal and MP j) on the different binding sites and k) with the different coverage of MP. l) Bond length changes between lattice Li and Li adatom in the presence of MP.

Three different concentrations of MP (200 ppm, 1, and 5 wt.%) were added to the electrolyte to determine the concentration effect of the MP molecular dipoles on the Li stripping and plating behavior. The electrolyte prepared with 200 ppm additive shows a relatively high cell overpotential of ≈300 mV as the cycling progressed, and voltage fluctuations were observed just after 120 h. When the concentration of MP was increased to 1 wt.%, the cell showed the most stable cycling performance with a low overpotential of ≈95 mV and exhibited flat and smooth voltage plateaus over 150 h. Interestingly, the Li–Li symmetric cell prepared with a higher MP concentration of 5 wt.% showed severe voltage fluctuations and a large overpotential when compared to the cells prepared using 200 ppm and 1 wt.% MP (**Figure** **3a**). While the MP dipoles flatten the deposited Li surface, an excess amount of adsorbed MP molecules on the Li reaction sites unexpectedly impede the Li deposition and stripping reactions, which induces a large voltage polarization. These results show it is important to identify the optimal concentration of MP additive used to successfully achieve stable Li metal reaction characteristics (Figure 3b). **Figure** **4c–e** shows the surface morphologies of the Cu foil surface after Li deposition using different MP concentrations in the electrolyte for 2 h at a current density of 0.5 mA cm^−2^ to directly verify the surface leveling effects with the concentration of MP used. The Li deposited on Cu foil using 200 ppm and 5 wt.% of the MP additive shows unfilled bumpy areas, in which a flat Li deposition process was not completed on the Li‐plated surface (Figure 3c,e). Furthermore, the significant growth of sharp and elongated needle‐like Li dendrites was observed in the enlarged images of the unfilled bumpy areas (see the insets of Figure 3c,e). However, the scanning electron microscopy (SEM) images of the Li surface deposited on the Cu foil using 1 wt.% of MP additive exhibited flatter and smoother surface features when compared to the other concentration conditions studied (Figure 3d). It was demonstrated that the Li deposition behavior could be effectively controlled and stabilized via appropriate optimization of the MP concentration.

### 2.3 Morphological Surface Leveling Effect of MP Molecular Dipoles

SEM observations were carried out after long term cycling (500 cycles) at a limited capacity of 0.5 mAh cm^−2^ and current density of 5 mA cm^−2^ in a Li–Li symmetric cell to clarify the surface leveling and stabilizing effect of the Li‐metal electrode upon introducing MP molecular dipoles (Figure 4a,b,d,e; Figure S3a,b, Supporting Information). Byproducts and the rough surface feature were observed on the plated Li‐metal electrode after cycling without the MP additive (Figure 4a; Figure S3a, Supporting Information). Many irregular pits were observed in the cross‐sectional images due to the non‐uniform and non‐homogeneous Li deposition and stripping processes (Figure 4b).^[^ ^14^ ^]^ As a result, the appearance of the pristine Li‐metal electrode observed after disassembling the Li–Li symmetric cell prepared without MP showed a dark and black surface, corresponding to dead Li or other unwanted side products (Figure 4c; Figure S3a, Supporting Information). In contrast, the surface morphology of the Li‐metal electrode cycled in the presence of the MP additive presents a relatively flatter and smoother surface without notable pits or debris (Figure 4d; Figure S3b, Supporing Information). The vertical image of the Li metal cycled with MP confirmed that the surface was formed smoothly with few pits (Figure 4e). The appearance of the Li metal disassembled from the cycled cell prepared with MP shows a shiny and silver color similar to the original features of Li metal with a negligible black edge surface (Figure 4f). SEM and optical observations of the cycled Li‐metal electrodes directly support the surface leveling and stabilizing effects of the MP molecular dipoles. In addition, atomic force microscopy (AFM) analysis of the cycled Li‐metal electrode prepared with and without MP was performed in an Ar‐filled glove box to investigate the surface and cross‐sectional features of the plated Li on the microscopic scale. Similar to the SEM results, a nodular surface with a much higher surface roughness (248.664 nm) was observed for the Li‐metal electrode cycled without MP (Figure 4g). For the Li‐metal electrode cycled in the presence of MP, we confirmed the surface leveling effect by observing a flat surface and much lower surface roughness (29.177 nm) (Figure 4h). By comparing the cross‐sectional heights of the Li‐metal electrode surfaces cycled with and without MP, it was confirmed that a further planarized Li‐metal electrode surface was achieved upon the addition of MP without any significant deviation in height when compared to the notable roughness of the Li‐metal electrode cycled without MP (Figure 4i; Figure S4, Supporting Information).

### 2.4 DFT Calculations of the Li‐metal Surface Reaction Employing MP Molecular Dipoles

Density functional theory (DFT) calculations were performed to understand the effects of the MP molecular dipole additives. According to our X‐ray diffraction (XRD) analysis (Figure S5, Supporting Information), which will be discussed in the next paragraph more specifically, we could observe a prominent (110) peak on the Li deposited with MP, but a (200) peak on the Li deposited without MP. We first checked the binding energies at the different binding sites to elucidate how the local atomic environment can affect the Li dendrite formation behavior. Three different binding sites, terrace, concave, and convex, were explored as possible defective nucleation sites. In the presence of MP, the binding energy becomes negative when compared to the positive energy of the Li binding sites in the absence of MP, which indicates that MP successfully stabilizes the binding sites and enhances the Li binding force. Li binding via the concave site can lead to horizontal Li growth or the (110) surface. However, Li binding via the convex or terrace sites can lead to the formation of Li dendrites via vertical growth. As expected, in the presence of MP, the concave site shows the strongest Li binding energy among the possible binding sites (Figure 4j). We confirmed that the defects (step or convex sites) on the Li anode were seeding points for Li‐dendrite formation. The effect of the MP additive was also studied by calculating the binding energy in the same way to that used for the Li adatoms. Interestingly, the binding energy of MP was always stronger than that of Li, regardless of the binding site, which means that MP fully covers the Li anode surface. Furthermore, we checked the change in the MP binding energy as the coverage increased, and the MP molecular dipoles had a stronger binding energy than that of Li at any coverage (Figure 4k). The bond lengths between the Li adatom and Li surface prepared with (purple) or without MP (red) were also examined. Figure 4l clearly shows that all of the bonds between the Li adatom and the Li surface were elongated via the adsorption of MP. This result implies that the presence of MP weakens the binding energy of Li and enables free Li diffusion even after nucleation. This eventually blocks Li dendrite formation via surface reconstruction of the plated Li components. Therefore, we elucidated the effect of the MP additive on Li dendrite formation using the binding energies with different coverages and bond length calculations. We confirmed that the Li adatom favored the convex site at which the Li adatom can grow vertically and that the MP molecular dipole additives always have a stronger binding energy than that of Li, which can fill all of the defect sites on the Li anode. Moreover, MP blocks Li dendrite formation via Li surface reconstruction.

### 2.5 SEI Components and Structural Growth Characteristics

**Figure 5:**
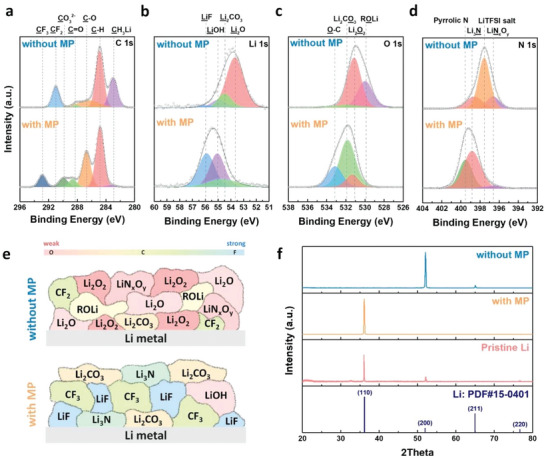
Ex situ measurements of the Li‐metal anode after 10 cycles. XPS spectra obtained from the charged Li‐metal electrode in an electrolyte with and without MP: a) C 1s b) Li 1s c) O 1s, and d) N 1s. e) A schematic illustration showing the composition of the SEI layer formed on the Li‐metal electrode surface. f) Ex situ X‐ray diffraction (XRD) peaks of the Li‐metal electrode obtained after the 10^th^ charge in an electrolyte with and without MP, and the pristine Li‐metal electrode.

To examine the chemical composition and surface structure of the SEI layers on the Li‐metal electrode, ex situ X‐ray photoelectron spectra (XPS) measurements of the Li electrodes with and without MP were conducted after 10 cycles. The C 1s, Li 1s, O 1s, and N 1s spectra obtained for the Li‐metal electrode were recorded (**Figure** **5**a–d). In the C 1s XPS spectrum, C‐H and C‐O peaks were mainly observed as a result of the decomposition of the TEGDME electrolyte (Figure 5a). The Li‐metal electrode cycled without the MP additive presents an additional strong CH_3_Li peak (282.4 eV) due to the side reaction of electrolyte decomposition,^[^ ^15^ ^]^ while a CF_2_ peak appeared for the Li‐metal electrode cycled without MP, and a CF_3_ peak appeared in the case of MP addition. According to a previous study, the coexistence of carbonate compounds, including fluorine and polymers, plays an important role in the formation of a flexible SEI layer.^[^ ^16^ ^]^ The CO_3_^2−^ peak (289.8 eV) becomes much stronger and broader in the presence of the MP additive, indicating that lithium carbonate products were dominant components in the SEI layer formed on the MP additive electrode surface. In the Li 1s spectra, a peak corresponding to Li_2_CO_3_ at 55 eV was also observed for both electrodes with and without MP (Figure 5b). The Li surface cycled without MP shows a strong Li_2_O peak (53.5 eV), a slight LiOH peak (55.2 eV), and small LiF peak (56 eV). However, a strong LiF peak at 56 eV was observed with a broad Li_2_CO_3_ peak at 55 eV in the presence of the MP additive. The XPS results demonstrate that the MP molecular dipoles help form a stable SEI layer, including durable components (e.g., fluorides and carbonates) on the Li metal surface.^[^ ^17^ ^]^ The LiF component in the SEI layer is well known as a Li‐protective component due to its low Li diffusion energy barrier, which enables homogeneous lithium‐ion flux and suppresses dendrite growth.^[8a,8b,18]^ In the O 1s spectrum obtained for the cycled Li electrode without the MP additive, two main peaks related to Li_2_O_2_ and LiOR were observed; the carbonate peaks were relatively marginal (Figure 5c). In contrast, the carbonate peaks of Li_2_CO_3_ and C—O as durable SEI components were observed in the presence of MP. Therefore, it was confirmed that the MP molecular dipoles in the electrolyte can change the internal composition of the SEI layer to mainly fluoride‐ and carbonate‐dominant phases instead of a weak oxide‐dominant phase, and to surface leveling effects, thereby working as a passivation layer to protect the Li‐metal electrode and prevent side reactions originating from electrolyte decomposition. In the N 1s spectra, a Li_3_N peak at 398.8 eV was observed in both electrodes with and without MP, and a relatively weak Li_3_N peak was observed in the absence of MP (Figure 5d). It can be seen that a useful SEI layer was formed in the presence of MP, in which Li _3_ N acts as a fast Li‐ion conductor, promoting Li‐ion diffusion and dissolving Li ions into free ions.^[^ ^19^ ^]^ Moreover, the bis(trifluoromethane) sulfonimide lithium salt (LiTFSI) peak (397.5 eV) caused by the salt contained in the electrolyte was very prominent in the absence of MP. On the other hand, the pyrrolic N peak was also observed at 399.6 eV, which is directly related to the MP molecules.^[^ ^20^ ^]^ Interestingly, our XPS results demonstrate another positive benefit in which the introduced MP molecules act as a forming agent to produce a stable and durable SEI layer on the Li‐metal electrode in addition to their surface leveling effect. Figure 5e illustrates the difference in the SEI layer elements formed with and without MP. The oxygen‐rich SEI phase (close to red) is weak and unstable against side reactions. The artificial formation of carbonate‐ and fluoride‐rich SEI phases (close to blue) is preferred to stabilize the Li‐metal electrode in harmful cell operating environments. While the Li‐metal electrode cycled without MP consists of weak oxygen‐rich SEI components, the MP molecular dipole helps form durable carbonate‐ and fluoride‐rich SEI phases for electrolyte decomposition. In addition, XPS depth analysis was performed on an electrode that had undergone 40 cycles to analyze the SEI chemical components that appear at different depths on the non‐uniform surface formed after long cycling. Figure S6 (Supporting Information) shows the results of the XPS depth analysis. In the absence of MP, the Li_2_O peak for O 1s increases noticeably with increasing etching time, and the peak intensities for F 1s are not uniform at different etch times. A cycled Li metal surface without MP shows the non‐uniform and weak SEI layer and the unstable Li_2_O component in the deep side of the SEI layer. On the other hand, a uniform and durable SEI layer is formed by the existence of MP without significant change.

Ex situ XRD analysis of the Li‐metal electrodes cycled with and without MP was used to further examine the changes in the crystalline structure of the Li‐metal electrode after cycling, as shown in Figure 5f. For pristine Li metal, two main peaks were observed and associated with the (110) and (200) planes of the cubic Li phase. After ten cycles in the absence of MP, the (110) peak disappears and the (200) peak becomes dominant, indicating that the crystalline structure of Li metal changes from its original structure as a result of the dendritic growth of Li and crystalline rearrangement. However, when the MP molecular dipole was used in the cycling process, the (110) peak was stably maintained without any peak shift observed after cycling. According to previous studies, the (110) peak of Li metal is related to a more stable crystal plane, corresponding to a low overpotential and stable Li surface formation.^[^ ^21^ ^]^ Interestingly, the (200) peak disappears in the case of the Li‐metal electrode cycled with MP and the intensity of the (110) peak increases further after cycling when compared to the pristine Li electrode. These results verify that the crystalline structure of the Li‐metal electrode was positively rearranged via Li plating and stripping in the presence of the MP molecular dipoles.

We can summarize the diverse advantages of the MP molecular dipoles as electrolyte additives for the stabilization of Li‐metal electrodes, as follows: i) The surface leveling effect that flattens the Li surface during Li plating and stripping, ii) durable SEI layer formation composed of carbonate and fluoride‐rich phases, and iii) crystalline structure rearrangement along the stable (110) plane after cycling. In this study, we have proposed a potential candidate to ensure the stabilization of Li‐metal electrodes by designing dipolar molecules based on surface leveling and stabilizing monomer agents, thereby realizing long‐lasting and safe Li‐metal batteries.

## 3 Conclusion

In summary, we have introduced MP molecular dipoles as surface‐flattening and interface‐stabilizing agents for long‐lifespan Li‐metal batteries. Homogeneous lithium‐ion deposition without dendrite growth was achieved by simply adding MP molecular dipoles to the electrolyte due to the polarized and electron‐rich functional groups in the MP molecules. The excellent electrochemical stability of the Li–Li symmetric cells over 300 h (>600 cycles) was demonstrated by employing MP molecular dipoles at a high current density of 5 mA cm^−2^. A reduced overpotential and flat voltage plateaus without voltage fluctuations were also achieved in the presence of MP. Reduced charge‐transfer resistance values of the cells with the MP molecules were observed during repeated cycling. We found that an optimal concentration of the MP additive is required to effectively stabilize the Li anode operation. We directly verified the surface‐flattening effect of the MP molecular dipoles using AFM. MP molecular dipoles help increase the Li binding energy via concave sites and facilitate horizontal and flattened Li growth along the (110) surface instead of Li binding via the convex or terrace sites related to vertical dendrite growth. We also confirmed that the chemical composition of the SEI layer formed in the presence of MP was close to a strong carbonate‐ and fluoride‐rich phase, rather than a weak oxygen‐rich SEI phase, thereby improving the surface stability of the Li‐metal electrode. The addition of multifunctional molecular dipole agents to effectively stabilize the Li‐metal anode offers a potential way to realize diverse Li‐metal battery systems.

## 4 Experimental Section

### Materials and Chemicals

Tetraethylene glycol dimethyl ether (TEGDME, 99%) was used as the electrolyte after drying for several days with freshly activated 4 Å molecular sieves. Bis(trifluoromethane) sulfonimide lithium salt (LiTFSI, 99.95%) was added to TEGDME and 1‐methyl‐2‐pyrrolidone (NMP, anhydrous, 99.5%) used as the electrolyte additive. All materials were purchased from Sigma‐Aldrich (Korea).

### Preparation of Li–Li Symmetric Cells

Li–Li symmetric cells were assembled using R2032 coin‐type cells (Wellcos Corp.) in an argon‐filled glovebox. A 12 mm diameter Li metal foil was used for both electrodes and Celgard 2500 polypropylene (PP) was used as the separator. 1 m LiTFSI in TEGDME with and without 1 wt.% MP was stirred for 24 h at room temperature and used as the electrolyte.

### Electrochemical Measurements

All electrochemical analyses were performed at room temperature. A charge/discharge cycle test using Li–Li symmetric cells was conducted using a battery cycler (WBCS3000S battery test system, WonATech). EIS measurements were performed from 1 MHz to 0.01 Hz with an amplitude of 5 mV.

### Ex situ Characterization

The surface morphologies were observed using field‐emission scanning electron microscopy (FE‐SEM, JSM‐7600F, JEOL) operated at an accelerating voltage of 5 keV. The surface roughness was investigated using AFM (NX‐10, Park Systems Korea) in a glove box. The surface atomic composition of the lithium metal electrodes after cycling was investigated using XPS (K‐alpha, Thermo U.K.). The crystal structure of the Li‐metal electrode was characterized using XRD (D8 Advance, Bruker) using Cu‐K*α* (*λ* = 1.54 Å).

### Computational Details

The Vienna ab initio simulation package (VASP) using a plane‐wave basis was used to perform spin‐polarized density functional theory (DFT) calculations. Electron exchange and correlation were considered by employing the revised Perdew–Burke–Ernzerhof (RPBE) functional^[^ ^22^ ^]^ widely accepted in surface chemical reaction studies.^[^ ^23^ ^]^ We set the cut‐off energy to 400 eV and sampled the k‐point mesh at 2 × 2 × 1 based on the Monkhorst–Pack scheme. The convergence criteria for electronic and geometry optimization were selected to be 10^−5^ eV and 10^−2^ eV Å^−1^, respectively.

The (110) surface of a Li BCC structure with defects (specifically, steps) was used to describe the actual Li‐metal anode structure. The Li slab model was centered between the 20 Å vacuum gap in the z‐direction and the bottom two atomic layers were fixed in their bulk positions. The binding energy of the Li atom (or the MP additive) was calculated using the following equation:

(1) $$\Delta E_{bind}=\;\Delta E_{Surface+Li\;\left(or\;MP\right)}-\;\Delta E_{surface}-\;\Delta E_{Li\;\left(or\;MP\right)}$$

where Δ*E_bind_* is the binding energy of the adsorbate, Δ*E*_*Surface* + *Li* (*or* *MP*)_ is the total energy of the Li surface with adsorbate, Δ*E_surface_* is the total energy of the isolated Li surface, and Δ*E*_*Li* (*or* *MP*)_ is the cohesive energy of the Li bulk or gas energy of the MP additive.

## Conflict of Interest

The authors declare no conflict of interest.

## Supporting information

Supporting Information

## Data Availability

The data that support the findings of this study are available from the corresponding author upon reasonable request.
