# Supplementary material for: Molecular Dipoles as a Surface Flattening and Interface Stabilizing Agent for Lithium‐Metal Batteries
Source: Adv Sci (Weinh). 2023 May 23;10(23):2301426. doi: 10.1002/advs.202301426 (PMC10427410; doi:10.1002/advs.202301426)
Supplement: Supplementary file 1 — Supporting Information [file ADVS-10-2301426-s001.pdf]

ADVANCED SCIENCE
Open Access

Supporting Information

for *Adv. Sci.*, DOI 10.1002/advs.202301426

Molecular Dipoles as a Surface Flattening and Interface Stabilizing Agent for Lithium-Metal Batteries

*Seo-Young Jun, Kihyun Shin, Jun-Seo Lee, Suji Kim, Jinyoung Chun and Won-Hee Ryu**

Supporting Information

Molecular Dipoles as a Surface Flattening and Interface Stabilizing Agent for Lithium-metal Batteries

Seo-Young Jun,[1] Kihyun Shin,[2] Jun-Seo Lee,[1] Suji Kim,[1] Jinyoung Chun,[3] and Won-Hee Ryu[1,3,]*

S. -Y Jun, J.-S. Lee, S. Kim, W.-H. Ryu

Dept. of Chemical and Biological Engineering, Sookmyung Women's University, 100 Cheongpa-ro 47-gil, Yongsan-gu, Seoul, 04310, Republic of Korea

E-mail: whryu@sookmyung.ac.kr

K. Shin

Dept. of Materials Science and Engineering, Hanbat National University, Daejeon 34158, Republic of Korea

J. Chun

Emerging Materials R&D Division, Korea Institute of Ceramic Engineering and Technology (KICET), Jinju 52851, Republic of Korea

W.-H. Ryu

Institute of Advanced Materials and Systems, Sookmyung Women's University, 100 Cheongpa-ro 47-gil, Yongsan-gu, Seoul 04310, Republic of Korea

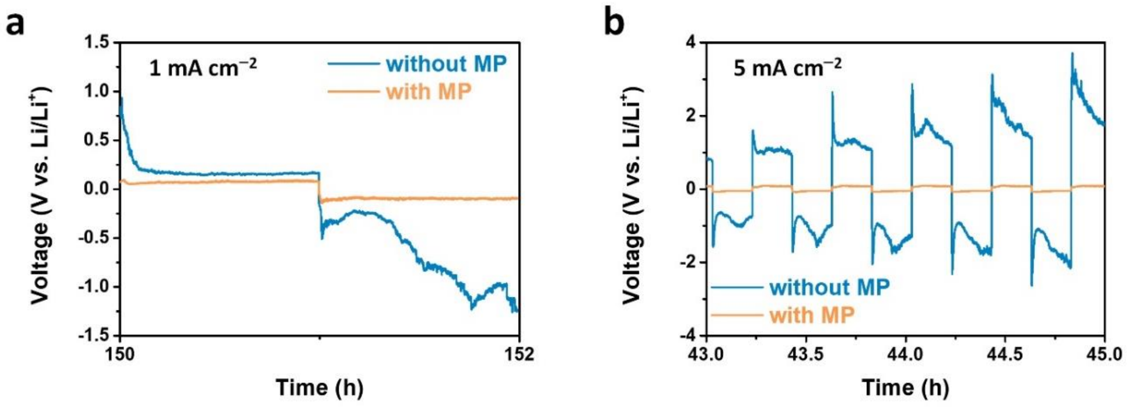

Figure S1. The partial detailed profiles of Figure 2a and c, which show the voltage plateau and overpotential.

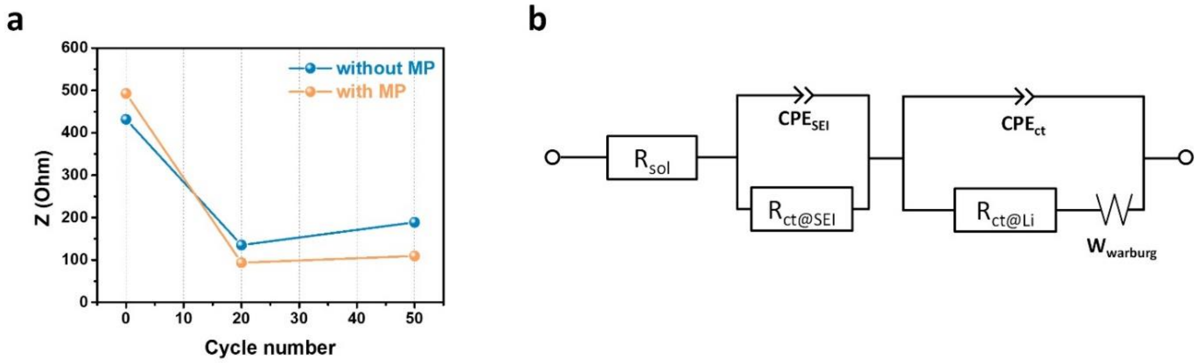

Figure S2. (a) Overall charge transfer resistance obtained from the Nyquist plots of both cells using the pristine electrolyte and electrolyte containing MP at different cycle numbers. (b) The equivalent circuit used for fitting

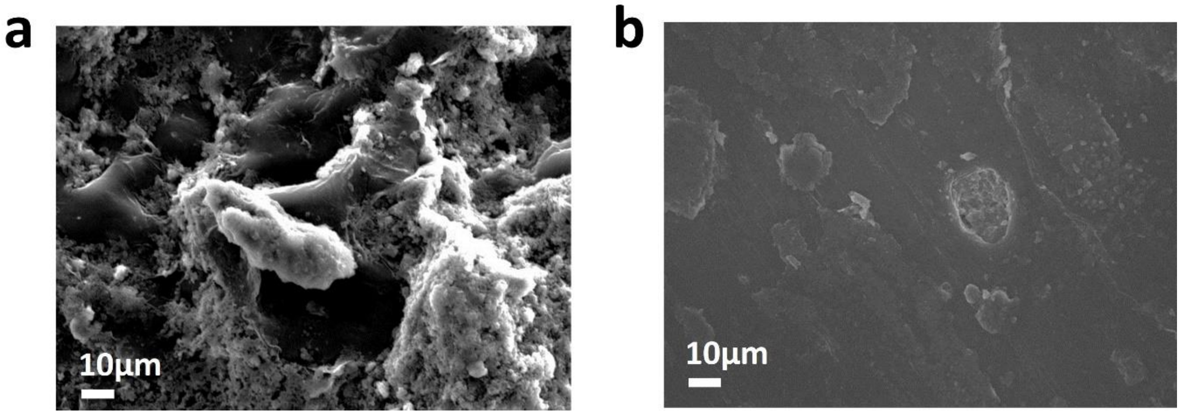

Figure S3. *Ex-situ* surface morphology of the Li-Li symmetric cell observed at a current density of 5 mA cm^{-2}. The SEM images of the Li-metal morphology after 500 cycles at a current density of 5 mA cm^{-2} in the electrolyte (a) without and (b) with MP at high magnification.

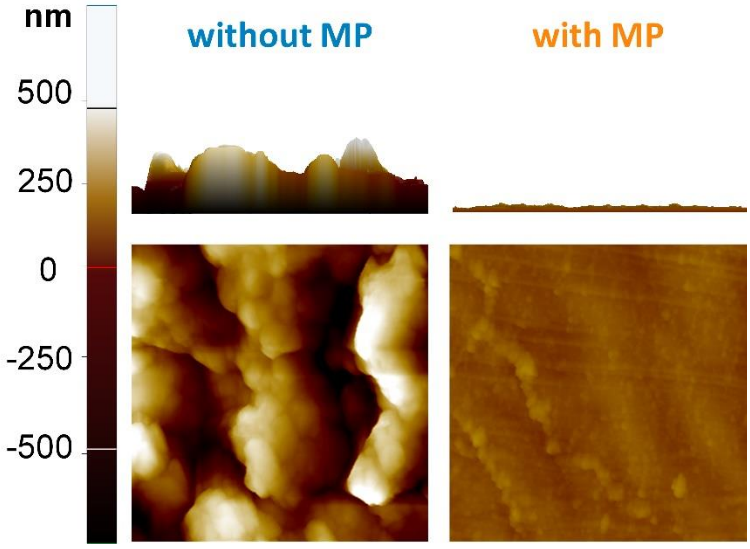

Figure S4. Cross-sectional images of the AFM measurements obtained with and without the MP additive.

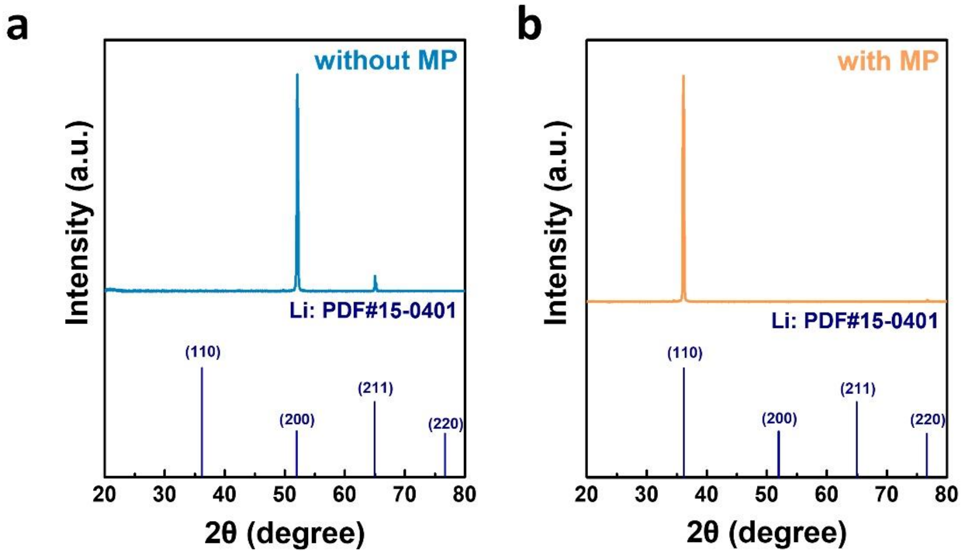

Figure S5. *Ex-situ* X-ray diffraction (XRD) patterns of the Li-metal electrode obtained after the 10th charge in the electrolyte (a) without and (b) with MP.

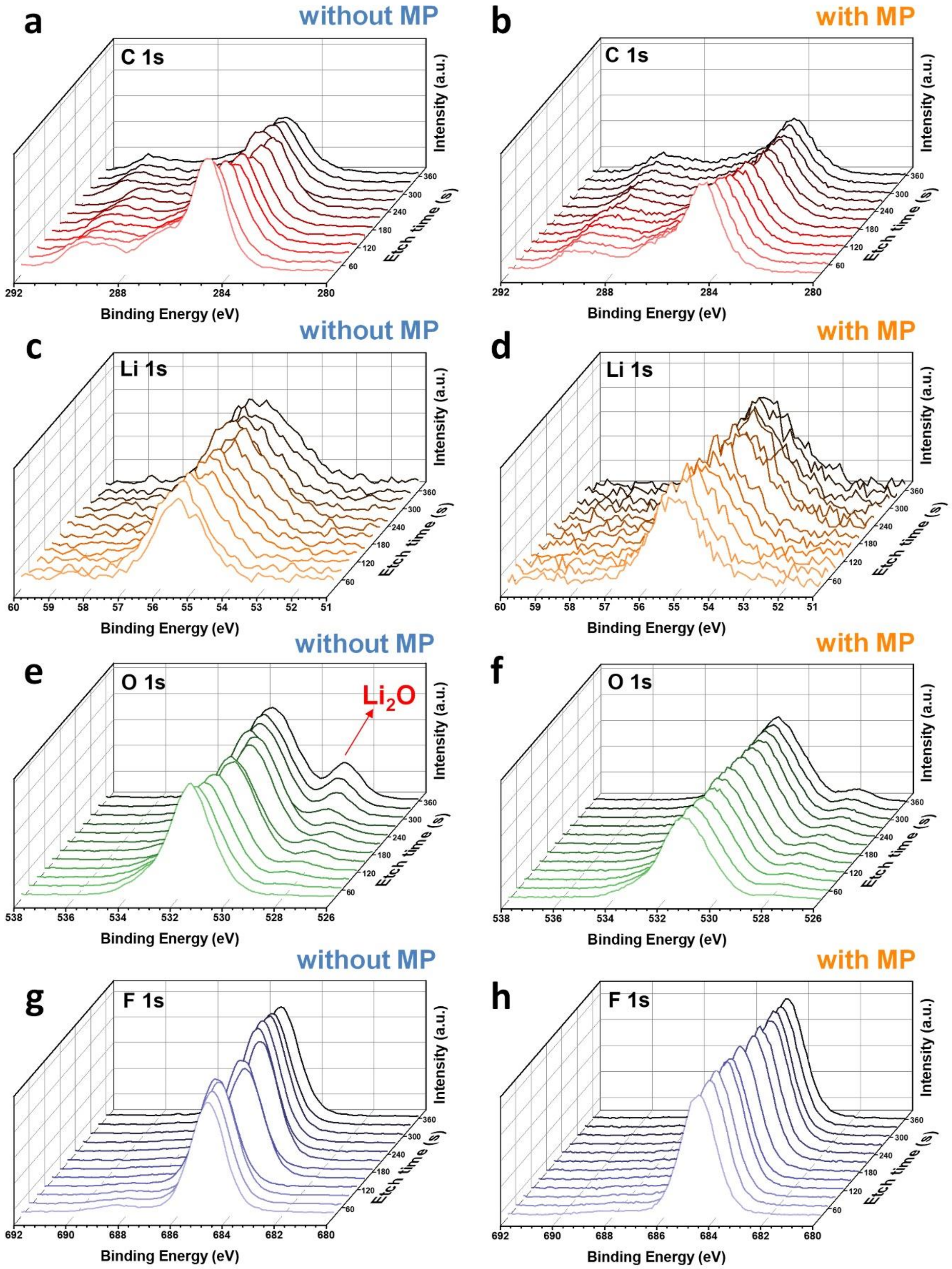

Figure S6. XPS depth profile results after 40 cycles. XPS depth profiles for (a) C 1s, (c) Li 1s, (e) O 1s, and (g) F 1s using electrolyte without MP, and (b) C 1s, (d) Li 1s, (f) O 1s, and (h) F 1s using electrolyte with MP additive.

	R_{sol}[Ω]		R_{ct}[Ω]	
	**without MP**	**with MP**	**without MP**	**with MP**
**Before cycle**	**7.02**	**5.38**	**438.79**	**498.05**
**After 20th cycle**	**10.85**	**5.57**	**146.03**	**99.05**
**After 50th cycle**	**22.22**	**17.24**	**211.16**	**126.73**

Table S1. The ohmic resistance and charge transfer resistance of the Li-Li symmetric cells prepared with and without MP additives after different numbers of cycles. The cycling tests were performed at a current density of 1 mA cm^{-2}.
